# Mortality Due to Chagas Disease in Brazil According to a Specific Cause

**DOI:** 10.4269/ajtmh.13-0574

**Published:** 2014-09-03

**Authors:** Aglaêr Alves da Nóbrega, Wildo Navegantes de Araújo, Ana Maria Nogales Vasconcelos

**Affiliations:** Tropical Medicine Center, University of Brasília, Brasília, Federal District, Brazil; Adjunct Professor of Post-Graduation in Tropical Medicine Center, University of Brasilia, Brasilia, Federal District, Brazil; Associate Professor of the Department of Statistics of University of Brasília, Brasília, Federal District, Brazil

## Abstract

A century after its discovery, Chagas disease (CD) is still considered a public health problem. Mortality caused by CD between 2000 and 2010 was described according to the specific underlying cause, year of occurrence, gender, age range, and region of Brazil. The standardized mortality rate decreased 32.4%, from 3.4% in 2000 to 2.3% in 2010. Most of the deaths (85.9%) occurred in male patients who were > 60 years of age caused by cardiac involvement. The mortality rate caused by cardiac involvement decreased in all regions of Brazil, except in the North region, where it increased by 1.6%. The Northeast had the smallest and the Central-West had the largest decrease. The mortality rate caused by a compromised digestive tract increased in all regions. Despite the control of transmission by vector and blood transfusions, CD should remain on the list of priority diseases for the public health service in Brazil, and surveillance actions cannot be interrupted.

## Introduction

Chagas disease (CD), or American trypanosomiasis, is considered by the World Health Organization (WHO) as a neglected tropical disease fostered by poverty^1^ and is a complex zoonosis caused by the protozoan *Trypanosoma cruzi.* In humans, the disease has an acute phase (which when detected, is a mandatorily reportable health condition in Brazil^2^) that may be asymptomatic or oligosymptomatic,^3^ and it progresses to a chronic phase, which may be indeterminate, cardiac, digestive, or mixed (simultaneously cardiac and digestive). Of the individuals affected by Chagas disease, between 20% and 40% have cardiac manifestations.^4^ Approximately 15–20% develop changes in the digestive tract, and more than 10% develop a mixed form of the disease.^1^,^5^,^6^

Almost all of the 8 million people infected with *T. cruzi* in 21 Latin America countries^7^ and 60 million are at risk of infection by *T. cruzi*.^8^ In Brazil there are about 2 to 3 million people infected with *T. cruzi*.^9^ There are also estimates that 5.4 million people will develop chronic heart disease and 900,000 will develop megacolon and megaesophagus.^10^

Since 2006, Brazil has been certified by the Pan American Health Organization as having eliminated the transmission of CD by *Triatoma infestans*, the main vector in the country. However, important host species of other potential transmission vectors are found in the North and Northeast regions of Brazil.^11^,^12^ Nevertheless, the number of people with the disease allows for the occurrence of new cases as a result of other forms of transmission; therefore, even if transmission stopped completely, the country would still have cases for many decades.^11^ Thus, we evaluated the trends in mortality caused by CD according to specific causes. These findings may contribute to the analysis of the control measures adopted and the health care and to the delineation of the disease burden in Brazil.

## Materials and Methods

### Type, population, and location of the study.

**Figure 1.**
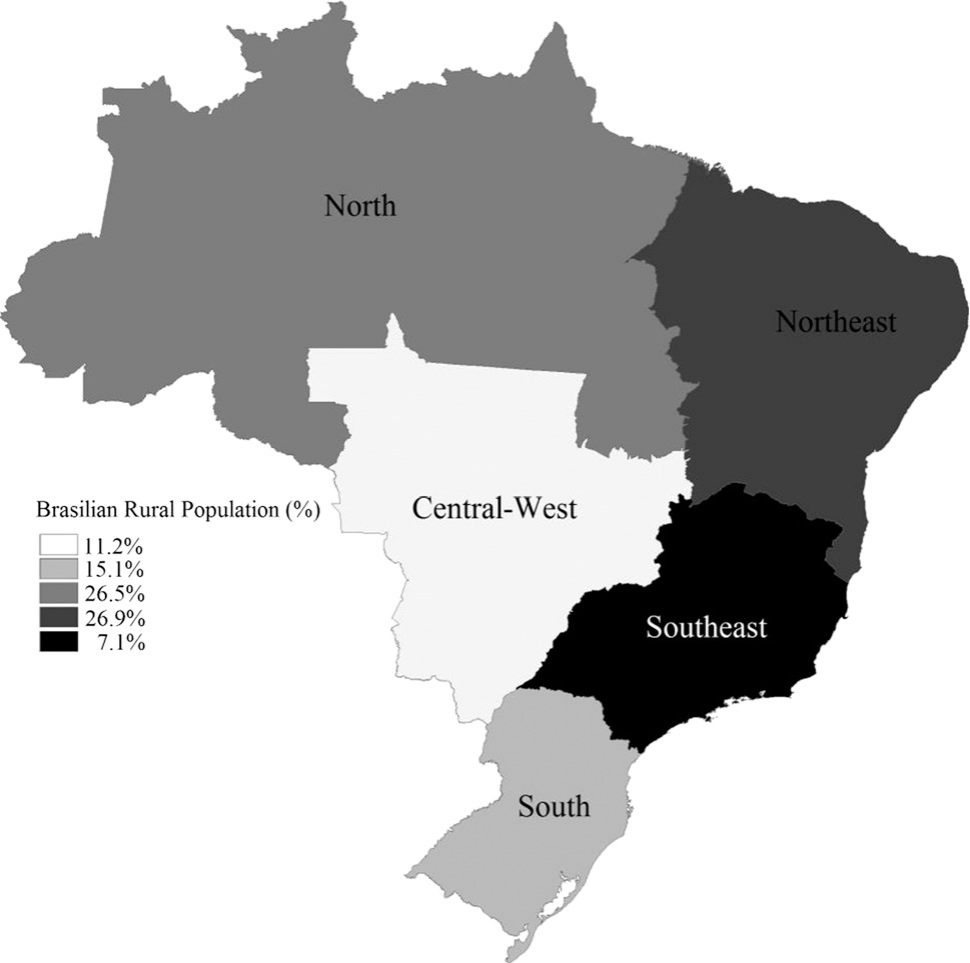
Map of Brazil showing the five geographic regions and percentage of Brazilian rural population in each one.

We conducted a descriptive study using data from all individuals who died of CD in Brazil between 2000 and 2010. Brazil has ∼200 million inhabitants, and 84% of them live in urban areas. The country is divided into five geopolitical regions (Figure 1), which have different demographic, economic, and social characteristics. The South, Southeast, and Central-West regions have lower illiteracy rates (4.9%, 5%, and 6.9%, respectively) and the highest per capita incomes. The North and Northeast regions have the highest illiteracy rates (11% and 18.5%, respectively) and the lowest per capita incomes.^13^

### Data source.

The number of deaths caused by CD according to underlying cause, year of occurrence, gender, age and region of Brazil were extracted from the Mortality Information System (Sistema de Informação sobre Mortalidade, SIM, in Portuguese), which is the official death registration system of the Brazilian Ministry of Health. In this system is unavailable data about mode of transmission and treatment used by each patient. It is an unique, standardized, and electronic database that contains the national public domain death records. The database was obtained from the website of the Department of Informatics of the Unified Health System (Sistema Único de Saúde, SUS), the DATASUS (http://tabnet.datasus.gov.br/tabdata/ sim/dados/cid10_indice.htm).

Since 1976, Brazil has used a single death certificate (DC) model throughout the national territory as the base document for the SIM.^14^ The DC contains demographic data (name, gender, age, race/color, education, place of residence, place of occurrence of death, etc.) and clinical information (underlying and associated cause) of the patient, and it is issued by the physician. Since 1996, the country has used the 10th version of the International Classification of Diseases (ICD-10) for coding the causes of death.

For the years 2000 and 2010, population data were obtained from the census data from the Brazilian Institute of Geography and Statistics.^15^ To obtain data for the period between the censuses, a linear interpolation was performed using the inter-census growth rate by age range, and the following age groups were adopted: 0–4, 5–14, 15–29, 30–44, 45–59, 60–69, 70–79, and 80 and older.^16^

### Data analysis and processing.

The coefficients of the specific mortality causes (per 100,000 people) were standardized and calculated by year, gender, age, and region of Brazil, using the direct method.^17^ The period analyzed was between 2000 and 2010, and the estimated Brazilian population for the year of 2005 was used as the standard. The causes of death for the B57 category (Chagas disease) were considered and comprised all subcategories (B57.0 to B57.5) defined by the ICD-10. The causes of death were grouped as follows: 1) acute and chronic CD with heart involvement (B57.0 and B57.2); 2) CD (chronic) with digestive system involvement (B57.3); 3) CD (chronic) with nervous system involvement (B57.4) and involvement of other organs (B57.5); and 4) acute CD without heart involvement (57.1). We compared the proportion of reduction of the mortality rates using the first (2000) and last (2010) year of the period studied.

This study was submitted to and approved by the Research Ethics Committee of the School of Medicine of the University of Brasília (Faculdade de Medicina da Universidade de Brasília) under No. 041/2011.

## Results

In the studied period, 54,236 deaths caused by CD were recorded, which corresponded to 0.5% (11.319.541 deaths) of all certifications of underlying causes. During the 11-year period, the standardized mortality rate was reduced by 32.4%, decreasing from 3.4 deaths per 100,000 inhabitants in 2000 to 2.3 in 2010.

**Figure 2.**
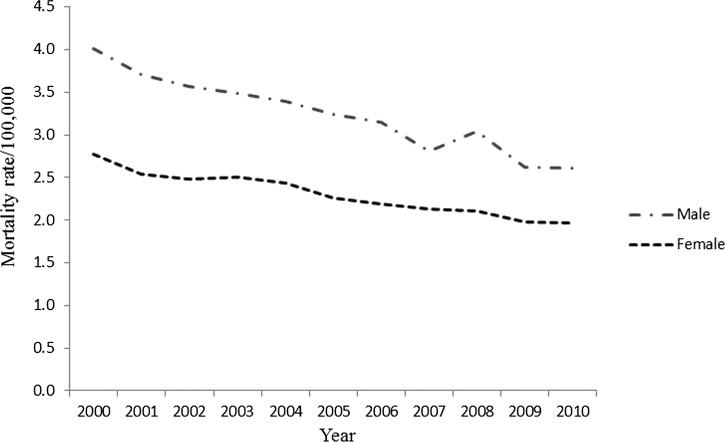
Mortality rates (per 100,000 people) caused by Chagas disease in Brazil, standardized by age, gender, and year of occurrence.

**Figure 3.**
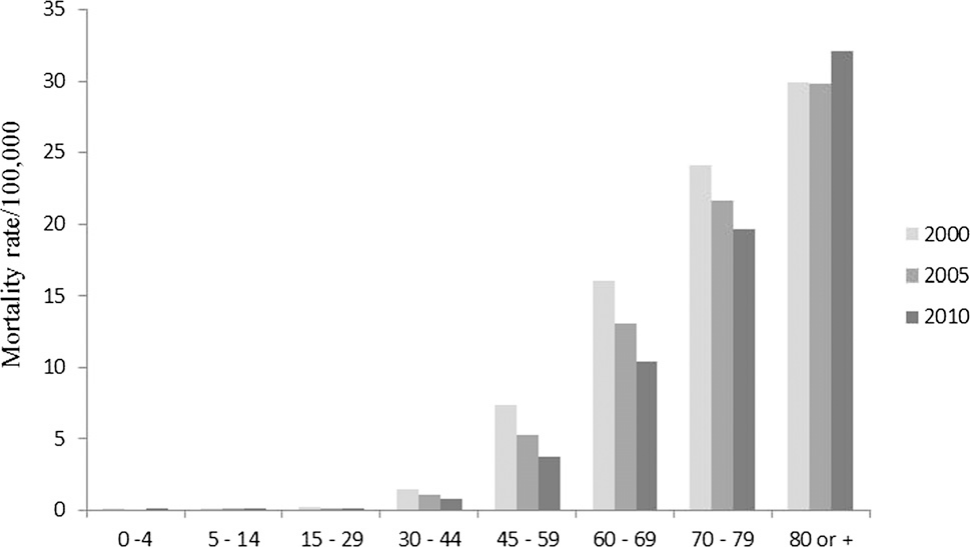
Standardized mortality rate caused by Chagas disease in Brazil according to age range and year of occurrence.

Most of the subjects (31,171 of 54,236; 57.5%) were male, and the mortality rates were higher among men than among women (Figure 2

). In both genders, a decline in the mortality rates was observed, being more pronounced in males (34.8%) than in females (28.9%). Sixty-three percent (34,165 of 54,236) of the patients were 60 years of age or more, and the mortality rate during the studied period decreased in all age ranges, except among those over 80 years of age (Figure 3

).

Cardiac involvement was responsible for 86% (46,581 of 54,236) of the deaths, and 97% (45,065 of 46,581) of these deaths occurred as a result of the chronic form of the disease. A compromised digestive system accounted for 11% (5,994 of 54,236) of the deaths. The involvement of other organs caused 2.6% (1,418 of 54,236) of the deaths, and the acute form of the disease was responsible for 0.4% (237 of 54,236) of the deaths. Among the study subjects that died, 52% (28,149 of 54,236) lived in the Southeast, 22% (11,847 of 54,236) lived in the Central-West, 19% (10,345 of 54,236) lived in the Northeast, 6% (3,119 of 54,236) lived in the South, and 1% (776 of 54,236) lived in the North.

**Figure 4.**
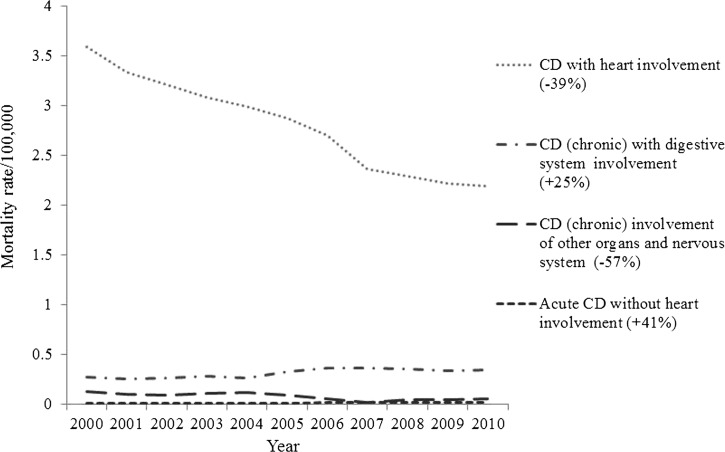
Standardized mortality rate (per 100,000 people) caused by specific cause in male gender in Brazil.

**Figure 5.**
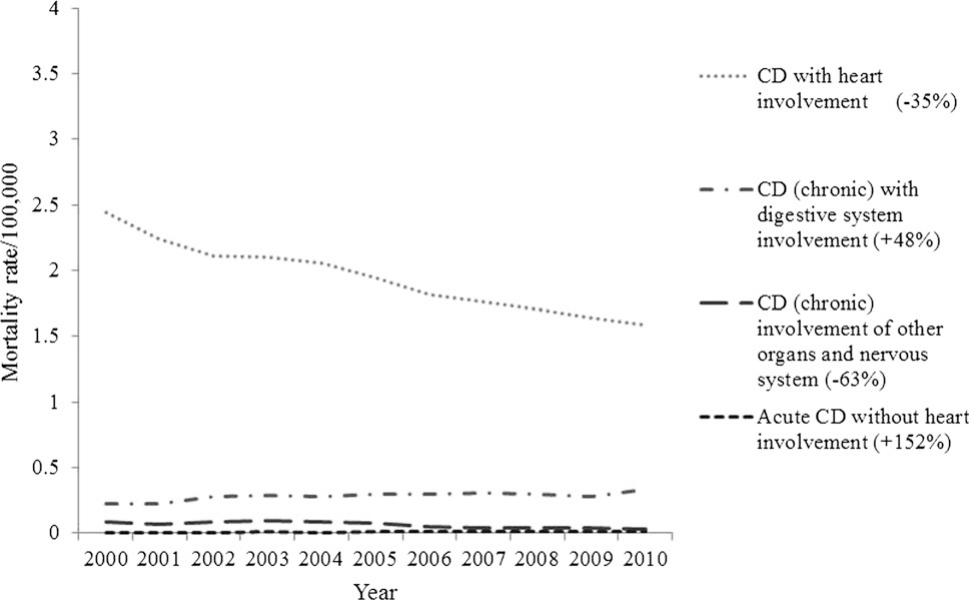
Standardized mortality rate (per 100,000 people) caused by specific cause in female gender in Brazil.

The mortality rates for CD caused by cardiac involvement and the involvement of other organs decreased during the studied period; however, the mortality rates as a result of a compromised digestive system and the acute form of the disease without cardiac involvement increased in male (Figure 4

) and in female genders (Figure 5

).

**Figure 6.**
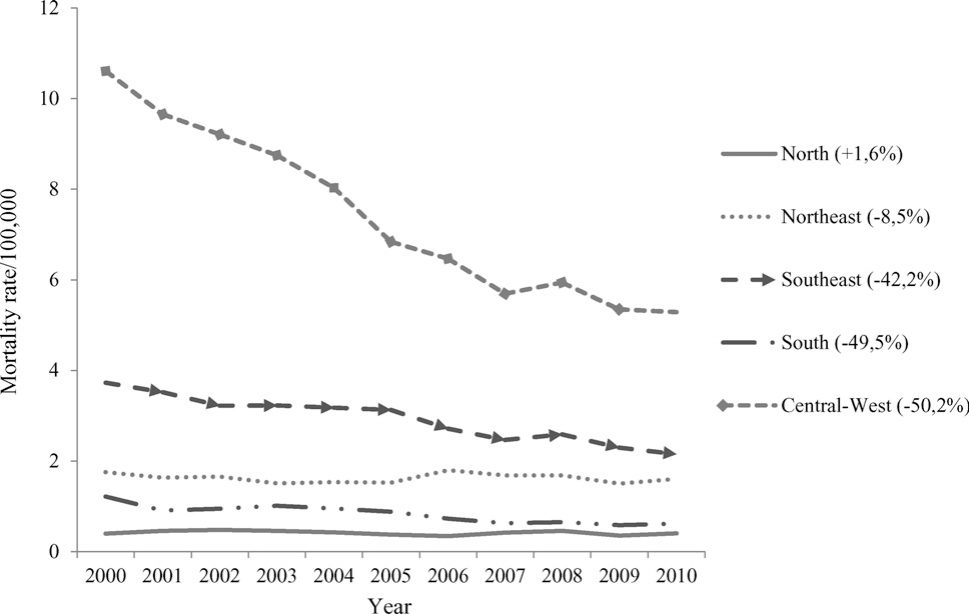
Standardized mortality rate (per 100,000 people) caused by cardiac involvement by region in Brazil.

The mortality rate caused by cardiac involvement decreased in all regions of Brazil, except in the North region, where it increased by 1.6%. The Northeast region had the smallest decrease (−8.5%) in mortality rate, and the Central-West showed the largest decrease, with a 50.2% reduction (Figure 6).

**Figure 7.**
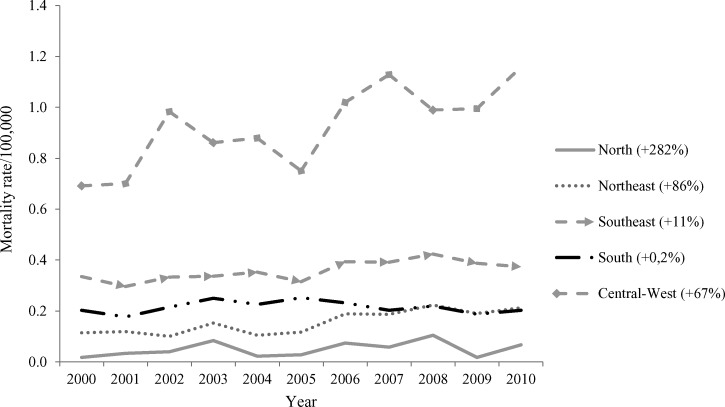
Standardized mortality rate (per 100,000 people) caused by digestive involvement by region in Brazil.

The mortality rate caused by a compromised digestive tract was increased in all regions, and the largest increases occurred in the North (+282%), Northeast (+86%), and Central-West (+67%) regions (Figure 7). Additional data are availabe in Supplemental Tables 1 and 2.

## Discussion

One century after its discovery,^18^ CD remains a public health issue of national importance. Considering the natural history of CD, when analyzing the age of individuals who died between 2000 and 2010, we speculated that they were infected between the 1960s and 1990s, when *T. infestans* was still the main vector of CD in Brazil, mainly in the Southeast, South, and Central-West regions,^12^ and a national serological survey for infection showed high (4.2%) prevalence of the disease in rural area.^19^ Population aging is leading to the occurrence of deaths at a more advanced age. The combined effect of reduced mortality and fertility rate has caused the aging index to increase 113% over 19 years.^20^ This fact may offer a possible explanation for the increased mortality rate among those > 80 years of age, assuming the same expectancy life in all countries. Furthermore, with the creation of the Unified Health System (SUS) in 1988, health services were expanded, allowing for performing early diagnosis and offering support treatments and more adequate follow-up, in addition to free treatment.^21^ There are only two drugs available for the treatment of CD: benznidazole and nifurtimox, which were developed nearly 40 years ago, have many side effects, and are effective in only some stages of the disease.^22^,^23^

In our study, cardiac involvement was responsible for most of the deaths in all regions of the country, although a histopathological study has shown the existence of strains of the parasite with tissue tropism for cardiac muscle in Central-West and for skeletal muscle in the South region of Brazil.^24^ Nonetheless, the mortality rate as a result of this cause has been decreasing in the country, except for the North region. Advances in the control of the disease, which can be observed in the reduced prevalence^25^ and risk for other forms of transmission, such as congenital and transfusion^26^ transmission, may have resulted from public investment. The actions resulting from this investment were aimed at improving housing in endemic areas for CD since 1967 to 1969^27^ and from the Southern Cone Initiative (of which Brazil is a signatory), whose objectives consisted of eliminating *T. infestans* infestations from houses and peridomestic environments in endemic areas, reducing and eliminating the infestation of other triatomine species in the same areas occupied by *T. infestans* and reducing and eliminating the transmission by blood transfusions.^28^ Other factors, such as the urbanization process of the country observed since the 1970s and the increase in the income of the population, may have contributed to this reduction.^21^

Nonetheless, the number of deaths caused by the digestive form of the disease has increased in all regions. We hypothesized that there are two possible explanations for this fact. The first hypothesis refers to the absence of a specific coding for the digestive component of CD in the 9th version of the International Classification of Diseases (ICD-9).^29^ With the adoption of ICD-10, which has a specific coding, the digestive component could be described on the death certificate and, therefore, had greater visibility. Additionally, the quality of the diagnosis was possibly improved by the available health technologies (laboratory diagnosis, etc.) and skilled health professionals, increasing its sensitivity and causing an increase in the number of cases of CD. Furthermore, the relative reduction in the mortalities caused by other forms may have given a greater visibility to the digestive form.

Two factors may have contributed to the increased mortality rate caused by CD in the North region of Brazil. The first factor is the increased coverage of the Mortality Information System records. From 1991 through 2010, the ratio between the number of reported and estimated deaths increased from 59.5 to 85.4.^13^

The second factor is the increased sensitivity of the health surveillance system. Although the presence of animal reservoirs and wild vectors of *T. cruzi* in the North region of Brazil has been well known since 1924,^30^ this area is considered non-endemic for CD, and the first autochthonous case of the disease was recorded only in 1969.^31^ However, further investigation of the disease began because the occurrence of acute cases caused by oral transmission had been acquiring importance in the region since 2000,^32^ and these events may have sensitized the surveillance system to record more deaths caused by CD, which were previously misdiagnosed. Nonetheless, because most of the deaths that occurred during the studied period were a result of the chronic form of the disease, it is possible that the individuals who died were those who had migrated from other regions of Brazil to the North in the 1970s and 1980s,^33^ as suggested by Drumond and Marcopito.^34^

The heterogeneity of the Mortality Information System coverage within the Brazilian regions may have underestimated the data presented. The North and Northeast regions have lower capacity of etiological diagnosis of the diseases and it has higher percentages about undefined causes in the database of the Mortality Information System.^35^ In addition, because these are secondary data, the existence of misclassified cases of the disease could not be evaluated.

Despite control of transmission by vectors, transfusions, and the reduced mortality caused by the cardiac form of the disease, CD should remain on the list of priority diseases for the public health service in Brazil because of its severity and economic burden.^36^,^37^ Therefore, surveillance and control actions cannot be interrupted, and the public and private pharmaceutical industries should be encouraged to develop new drugs to treat the different phases of the disease.

## Supplementary Material

Supplemental Tables.
