# Supplementary material for: Mortality Due to Chagas Disease in Brazil According to a Specific Cause
Source: Am J Trop Med Hyg. 2014 Sep 3;91(3):528–33. doi: 10.4269/ajtmh.13-0574 (PMC4155553; doi:10.4269/ajtmh.13-0574)
Supplement: Supplementary file 1 [file SD4.pdf]

Supplemental Table 1

Epidemiological aspects and mortality rate about the 237 deaths recorded as Chagas' disease acute form in the Mortality Information System, Brazil. 2000–2010

Epidemiological aspects	*N*	%	Mortality rate*
Gender			
Male	131	55	0.01
Age range			
0–4	1	0.4	0.00
5–14	1	0.4	0.00
15–29	6	2.5	0.00
30–44	26	11.0	0.01
45–59	59	24.9	0.02
60–69	53	22.4	0.05
70–79	52	21.9	0.08
80 or +	39	16.5	0.14
Region of Brazil			
North	7	2.9	0.00
Northeast	65	27.4	0.01
Southeast	115	48.5	0.01
South	19	8.0	0.01
Central-West	31	13.2	0.02

*Calculated by mean of the mortality rate (×100,000 inhabitants) using the deaths recorded as numerator and the estimative and census (2000 and 2010) of the population as denominator for the period studied.

Supplemental Table 2

Standardized mortality rate (per 100,000 people) caused by cardiac and digestive involvement by region in Brazil

	2000		2001		2002		2003		2004		2005		2006		2007		2008		2009		2010	
Region	CI*	DI†	CI	DI	CI	DI	CI	DI	CI	DI	CI	DI	CI	DI	CI	DI	CI	DI	CI	DI	CI	DI
North	0.40	0.02	0.46	0.03	0.48	0.04	0.46	0.08	0.42	0.02	0.37	0.03	0.34	0.07	0.42	0.06	0.45	0.10	0.35	0.02	0.40	0.07
Northeast	1.75	0.11	1.63	0.12	1.65	0.10	1.51	0.15	1.53	0.10	1.52	0.12	1.79	0.19	1.68	0.19	1.68	0.22	1.50	0.19	1.60	0.21
Southeast	3.72	0.34	3.51	0.30	3.22	0.33	3.22	0.34	3.17	0.35	3.13	0.31	2.71	0.39	2.47	0.39	2.58	0.42	2.29	0.39	2.15	0.37
South	1.21	0.20	0.90	0.18	0.94	0.22	1.01	0.25	0.95	0.23	0.88	0.25	0.73	0.23	0.63	0.20	0.65	0.22	0.58	0.19	0.61	0.20
Central-West	10.61	0.69	9.65	0.70	9.20	0.98	8.75	0.86	8.02	0.88	6.83	0.75	6.46	1.02	5.69	1.13	5.93	0.99	5.34	1.00	5.28	1.16

*Cardiac involvement.
†Digestive involvement.
